# Increased Genetic Diversity of HIV-1 Circulating in Hong Kong

**DOI:** 10.1371/journal.pone.0012198

**Published:** 2010-08-16

**Authors:** Jonathan Hon-Kwan Chen, Ka-Hing Wong, Zhiwei Chen, Kenny Chan, Ho-Yin Lam, Sabrina Wai-Chi To, Vincent Chi-Chung Cheng, Kwok-Yung Yuen, Wing-Cheong Yam

**Affiliations:** 1 Department of Microbiology, Queen Mary Hospital, The University of Hong Kong, Hong Kong, Special Administrative Region, China; 2 AIDS Institute, The University of Hong Kong, Hong Kong, Special Administrative Region, China; 3 Integrated Treatment Centre, Special Preventive Programme, Centre of Health Protection, Department of Health, Hong Kong, Special Administrative Region, China; Indiana University, United States of America

## Abstract

HIV-1 group M strains are characterized into 9 pure subtypes and 48 circulating recombinant forms (CRFs). Recent studies have identified the presence of new HIV-1 recombinants in Hong Kong and their complexity continues to increase. This study aims to characterize the HIV-1 genetic diversity in Hong Kong. Phylogenetic analyses were performed by using HIV-1 *pol* sequences including protease and partial reverse transcriptase isolated from 1045 local patients in Hong Kong from 2003 to 2008. For the *pol* sequences with unassigned genotype, the evidence of recombination was determined by using sliding-window based bootscan plots and their *env* C2V3 region were also sequenced. Epidemiological background of these patients was further collected. The *pol* phylogenetic analyses highlighted the extent of HIV-1 genetic diversity in Hong Kong. Subtype B (450/1045; 43.1%) and CRF01_AE (469/1045; 44.9%) variants were clearly predominant. Other genotypes (126/1045; 12.1%) including 3 defined subtypes, 10 CRFs, 1 unassigned subtype and 33 recombinants with 11 different mosaic patterns were observed. Recombinants of subtype B and CRF01_AE were mainly found among local Chinese MSM throughout 2004 to 2008, while the CRF02_AG and subtype G recombinants were circulating among non-Chinese Asian population in Hong Kong through heterosexual transmission starting from 2008. Our study demonstrated the complex recombination of HIV-1 in Hong Kong and the need in developing surveillance system for tracking the distribution of new HIV-1 genetic variants.

## Introduction

Human immunodeficiency virus (HIV) carries an error prone reverse transcriptase which causes its extraordinary genetic diversity [1]. Throughout the epidemic, 9 subtypes (A–D, F–H, J and K) and a further 48 major circulating recombinant forms (CRFs) has been documented within HIV type 1 (HIV-1) M group (http://www.hiv.lanl.gov/content/hiv-db/CRFs/CRFs.html). Genetic founder effect causes the heterogeneous and specific global geographic distribution of different HIV-1 group M strains [2]. HIV-1 subtype C infection is predominant in the world while the epidemic in the western world is primarily caused by HIV-1 subtype B variants [3]. In the East and Southeast Asia, CRF01_AE is highly predominant.

Meanwhile, the frequent self-recombination and ongoing exchange of HIV-1 strains between geographic regions through population migration and travel caused a small number of group M strains to move into new host populations [2]. In recent years, growing numbers of HIV-1 recombinant forms have been identified in different geographical regions [2]. The CRF01_AE, CRF07_BC, CRF08_BC, CRF33_01B and CRF34_01B were confirmed to be originated in Asia while new unique recombinant forms (URFs) are continually being identified in these years and their complexity continues to increase [4], [5], [6]. These URFs may later become new CRFs after circulating in particular geographical areas.

Up to the end of 2006, the HIV-1 subtype B (36.4%) and CRF01_AE (48.8%) are co-predominant in Hong Kong [7], [8]. Through phylogenetic estimation, the divergence date of these strains in Hong Kong is around 1995–2001 [9], [10]. However, the prevalence of other new recombinant forms in Hong Kong remains unclear. Hong Kong is a metropolitan city located at the centre of the Southeast Asia. The high migration and traveling population of Hong Kong with other HIV-1 epidemic Asian countries such as Mainland China, Thailand, India, Malaysia, etc. may enhance the development of new HIV-1 recombinants in the Southeast Asia.

As the rising number of new recombinant lineages can significantly impact therapeutics and vaccine development [11], [12], this study aims to investigate the prevalence and characterization of new recombinants circulating in Hong Kong from 2003 to 2008. Also, we would like to track the epidemiological transmission reservoir of these recombination variants.

## Results

### Distribution of HIV-1 group M variants in Hong Kong

**Figure 1:**
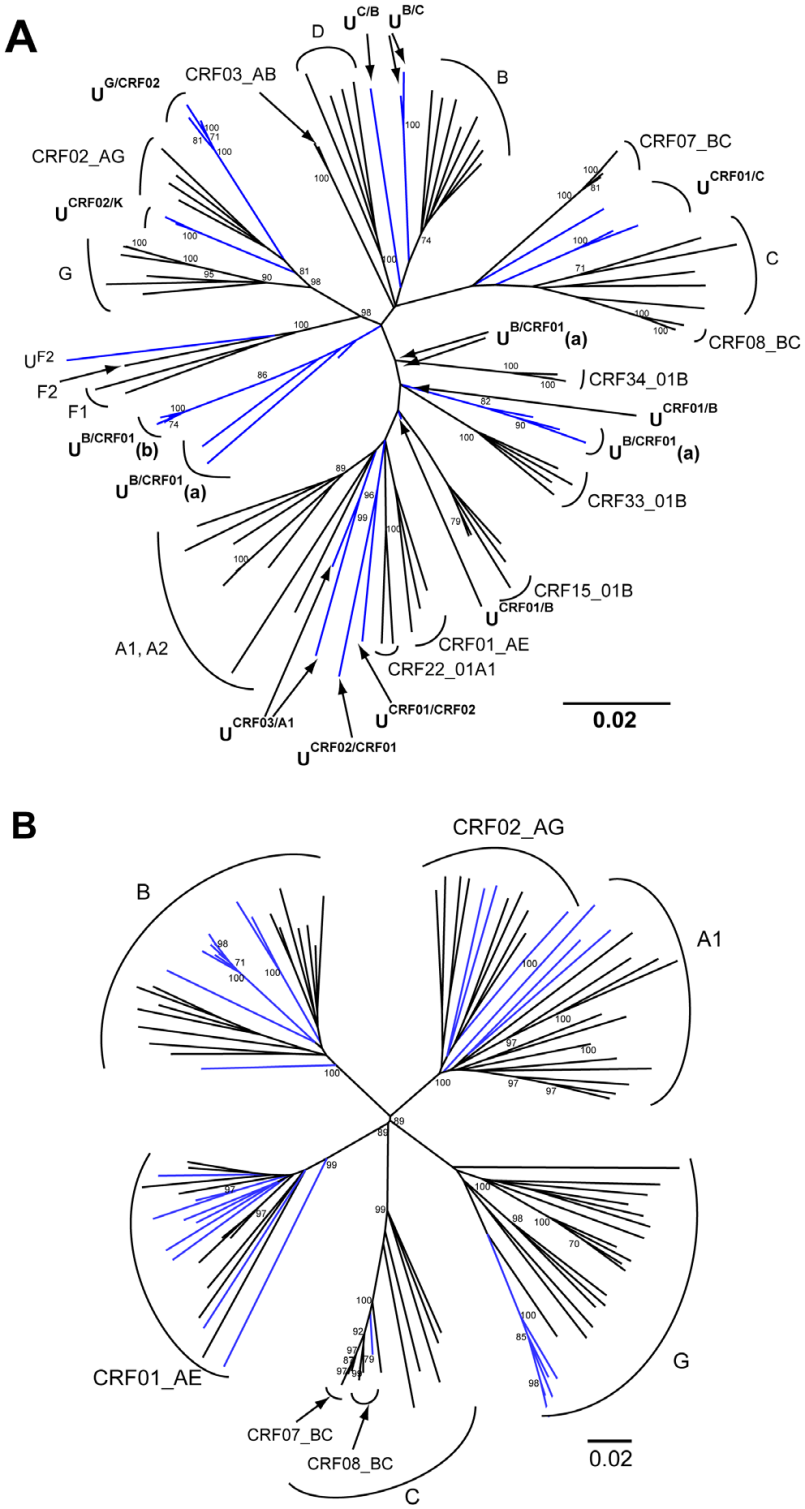
Phylogenetic relationships of 34 unassigned *pol* gene sequences (1a) and 31 unassigned *env* gene C2V3 region sequences (1b). Genotype unassigned sequences were highlighted in blue and all reference sequences were in black. Bootstrap values >70 were shown at the nodes of the trees.

The 1045 individuals were recruited in the Integrated Treatment Centre of the Department of Health from January 2003 to December 2008 which represented 51.4% (1045/2032) of all new diagnoses of HIV-1 infection in Hong Kong within that period of time (available at http://www.info.gov.hk/aids). The *pol* phylogenetic analysis could assign confident genotypes to 96.3% of samples (1006/1045), while subtype B and CRF01_AE accounted for 43.1% (450/1045) and 44.9% (469/1045) of the total number of samples respectively. HIV-1 variants with other genotypes were recognized in 12.1% of samples (126/1045) including subtype A1 (1/126; 0.8%), C (44/126; 34.9%), D (1/126; 0.8%), CRF02_AG (7/126; 5.6%), CRF03_AB (2/126; 1.6%), CRF06_cpx (1/126; 0.8%), CRF07_BC (20/126; 15.9%), CRF08_BC (9/126; 7.1%), CRF15_01B (2/126; 1.6%), CRF22_01A1 (1/126; 0.8%), CRF33_01B (2/126; 0.8%), CRF42_02G (1/126; 0.8%) and CRF43_02G (1/126; 0.8%). There were another 34 samples (34/126; 27.0%) that could not be confidently assigned to previously defined subtypes or CRFs by the *pol* phylogenetic analysis (Figure 1a).

The *env* C2V3 region of the 34 unassigned samples were further sequenced and confidently assigned genotypes (bootstrap value >70) could only be found on 31 samples in the *env* phylogenetic tree (Figure 1b) and 3 samples were failed in the *env* gene PCR-sequencing. There was no genetic recombination found in any of the 31 available *env* sequences while 1 sample was genotyped as subtype F2-like outgroup by using *pol* gene and CRF02_AG by using *env* gene (Table 1).

**Table 1:** The *pol* and *env* genotype of the 34 unassigned genotype samples.

*pol*	n	*env*	n
U^B/CRF01^	14	B	9
		CRF01_AE	4
		N/A	1
U^G/CRF02^	5	G	5
U^CRF01/C^	3	CRF01_AE	2
		A1	1
U^B/C^	2	B	2
U^CRF02/CRF01^	1	N/A	1
U^CRF01/CRF02^		CRF02_AG	1
U^CRF02/K^	2	CRF02_AG	2
U^CRF01/B^	2	CRF01_AE	1
		N/A	1
U^CRF03/A1^	2	A1	2
U^C/B^	1	CRF08_BC	1
U^F2^	1	CRF02_AG	1
Total	**34**		**34**

N/A represents PCR amplification or DNA sequencing failed.

**Figure 2:**
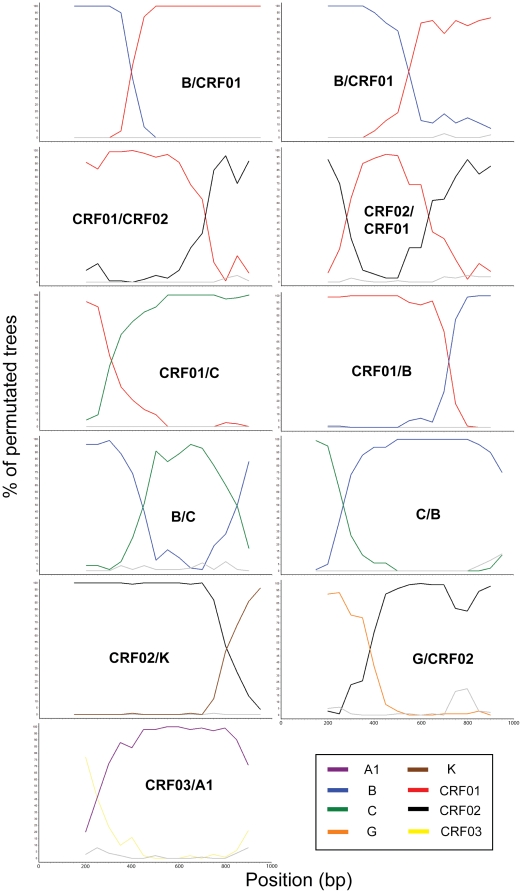
Bootscanning plots of the unassigned *pol* gene variants. The bootstrap values are plotted for a window of 300–400bp moving in increments of 50 bps along the alignment.

Through the bootscanning recombination analysis, 33 out of the 34 unassigned samples were found to have genetic recombination in the *pol* gene and another one was classified as unassigned subtype F2 (U^F2^). The bootscanning recombination patterns were shown in Figure 2. Samples with subtype B and CRF01_AE recombination in the *pol* region were identified in 16 samples with different recombination patterns (U^B/CRF01^(a), U^B/CRF01^(b) and U^CRF01/B^). Recombination of subtype G and CRF02_AG in the *pol* gene (U^G/CRF02^) were identified in another 5 samples. Three samples were found to have subtype B and subtype C recombination in the *pol* region with 2 different recombination patterns (U^B/C^ and U^C/B^), while another 3 samples were CRF01_AE and subtype C recombinants (U^CRF01/C^). Recombination of CRF02_AG and subtype K (U^CRF02/K^) (2 samples), CRF02_AG and CRF01_AE (U^CRF02/CRF01^ and U^CRF01/CRF02^) (2 samples), and CRF03_AB and A1 (U^CRF03/A1^) (2 samples) were also observed.

### Characteristics of new recombinant infected patients

The first recombinant sample in this study was collected in 2004 and the number of cases increased progressively in the following years (Table 2).

**Table 2:** The epidemiological background of the 33 genotype unassigned recombinants.

Category	*pol* gene recombination	Number of samples	Ethnicity and gender (transmission route)	Sampling year
U^B/CRF01^(a)	B/CRF01_AE	10	9 Chinese males (6 MSM and 2 heterosexual)1 Vietnamese male (IDU)	2004, 2005, 2006, 2007, 2008
U^B/CRF01^(b)	B/CRF01_AE	4	4 Chinese males (3 MSM and 1 heterosexual)	2005, 2007
U^G/CRF02^	G/CRF02_AG	5	2 Filipino females (heterosexual)1 Indonesian female (heterosexual)1 Bangladeshi male (heterosexual)1 Ghanaian male (heterosexual)	2008
U^CRF01/C^	CRF01_AE/C	3	1 Chinese female (heterosexual)1 Nepalese male (heterosexual)1 Nepalese female (heterosexual)	2006, 2008
U^CRF01/B^	CRF01_AE/B	2	1 Chinese male (heterosexual)1 Filipino female (heterosexual)	2006, 2007
U^B/C^	B/C	2	2 Chinese males (1 MSM and 1 heterosexual)	2004, 2006
U^CRF02/K^	CRF02_AG/K	2	1 Nigerian male (heterosexual)1 Filipino female (heterosexual)	2007, 2008
U^CRF03/A1^	CRF03_AB/A1	2	1 Chinese male (heterosexual)1 British male (heterosexual)	2006, 2007
U^CRF01/CRF02^	CRF01_AE/CRF02_AG	1	1 Chinese male (heterosexual)	2004
U^CRF02/CRF01^	CRF02_AG/CRF01_AE	1	1 Nigerian male (heterosexual)	2005
U^C/B^	C/B	1	1 Chinese male (heterosexual)	2004

The U^B/CRF01^ recombinant was the only recombination variant that was found every year between 2004 and 2008, while almost all these samples were collected from Hong Kong Chinese male residents (13 out of 14). The transmission route among these Chinese males was found to be MSM (9 samples) and heterosexual (3 samples) transmission. Another sample was isolated from a male Vietnamese immigrant with intravenous drug use. For the U^CRF01/B^ recombinant, it was isolated from a Chinese heterosexual male and a Filipino female. The Filipino female was confirmed catching disease through heterosexual transmission.

Another 5 U^G/CRF02^ recombinant cases were all isolated in 2008. This recombinant variant infected 2 non-Chinese male patients including 1 African from Ghana and 1 Bangladeshi. The other 3 patients were non-Chinese Asian females from Indonesia (1 patient) and the Philippines (2 patients). All 5 patients arrived in Hong Kong since 2006 with HIV infection through heterosexual transmission between 2007 and 2008.

Recombinant of subtype B and C (U^B/C^ and U^C/B^) were isolated from 3 Chinese males. The U^C/B^ infected patient was confirmed catching HIV-1 away from Hong Kong through heterosexual transmission.

The U^CRF01/C^ recombinants were identified in 3 heterosexual patients including 1 Chinese female, 1 Nepalese male and 1 Nepalese female. The two Nepalese were husband and wife while the wife had been confirmed to catch disease from the male patient.

For the U^CRF02/K^ recombinants, cases were reported in 2007 and 2008 which samples were collected from 1 Nigerian male and 1 Filipino female.

The U^CRF03/A1^ recombinant was identified in another 2 patients who got infection through heterosexual transmission (1 Chinese male and 1 British Caucasian male) while the U^CRF02/CRF01^ recombinant was identified in one Nigerian female and the U^CRF02/CRF01^ recombinant was found in one Chinese male.

## Discussion

Prior HIV-1 molecular epidemiology studies performed in Asia were mainly focused on the transmission of locally predominant HIV-1 genotypes [7], [10], [13], [14]. There was limited number of studies which focused on new HIV-1 group M recombinant transmission. In this study, we demonstrated the broad HIV-1 genetic diversity in Hong Kong and it is the first comprehensive study in Asia revealing the transmission of different new recombinants in the same locality.

The treatment naïve HIV-1 samples in this study were collected from 2003 to 2008 in Hong Kong and the study cohort herein was estimated to represent about 50% of the total HIV-1 patients in Hong Kong within the 5 years [15]. Among the total HIV-1 patients in Hong Kong, about 60–70% of them were local Chinese inhabitants, while the other 30–40% of patients was non-Chinese residents including non-Chinese Asians, Caucasians and Africans. From the large sample size included in this study, we can further confirm that subtype B (43.1%) and CRF01_AE (44.9%) were co-dominant HIV-1 genotypes among the local Chinese patients in Hong Kong. This genotyping distribution showed complete concordance to our recent studies [7], [10]. Other than these two predominant genotypes, another 12 defined subtypes or CRFs were also identified, while subtype C and CRF07_BC were more commonly found. Other defined HIV-1 CRFs, such as CRF22_01A1, CRF42_02G and CRF43_02G were sporadically found in Hong Kong and these CRFs are first ever reported in Hong Kong and China. This broad range of defined HIV-1 genotypes suggested the multiple route of transmission for the import of new HIV-1 genotypes into the Hong Kong local population.

On the other hand, our study also revealed the circulation of certain number of new HIV-1 recombinants among local HIV-1 patients in Hong Kong while the total number of HIV-1 infection is low throughout the past 10 years. Through the bootscanning recombinant analysis, 11 different mosaic *pol* genotypic patterns were found among the 33 unassigned recombinant samples. The U^B/CRF01^ and U^G/CRF02^ recombinant were further found to have local transmission among the Chinese MSM and the non-Chinese population in Hong Kong respectively. The U^B/CRF01^ recombinant cases were identified every year from 2004 to 2008 which suggested a stable transmission route of the U^B/CRF01^ recombinant has been established among the local Chinese MSM population. The presence of the U^B/CRF01^ recombinant in this patient risk group suggested the support the “genetic founder effect” hypothesis of the recombination between local subtype B and CRF01_AE HIV strains. By comparing the mosaic fractions of the new U^B/CRF01^ recombinants with other Asian *pol* sequences, the new U^B/CRF01^ recombinant is 97% identical to the circulating local CRF01_AE or pure subtype B viruses in Hong Kong. This suggests that local subtype B and CRF01_AE were origins of the U^B/CRF01^ recombinant. Full viral genome sequencing will be done to elucidate the sequences of this new recombinant.

This study also identified 5 cases of U^G/CRF02^ recombinant infection within 5 months in 2008. The patients were all non-Chinese Asian and African who arrived to Hong Kong starting from 2006. They were estimated to catch HIV-1 infection through heterosexual transmission between 2007 and 2008. As subtype G and CRF02_AG were not commonly found in Hong Kong in the past 10 years, we believe this U^G/CRF02^ recombinant is not originated from the local circulating subtype G and CRF02_AG strains. Since the viral *pol* sequences of the 5 U^G/CRF02^ cases demonstrated a 98% similarity, it is plausible that there might be linkages between the sources of infection with this recombinant strain in these non-Chinese cases. Although this study could not prove the origin of this new U^G/CRF02^ recombinant, there is high possibility that the U^G/CRF02^ recombinant was imported to Hong Kong between 2007 and 2008 through non-Chinese HIV-1 carriers.

In conclusion, our study revealed the broad genetic diversity of HIV-1 in Hong Kong and identified two local spreading new recombinant lineages. Since the rising number of new recombinant lineages can significantly impact therapeutics and vaccine development, therefore, a good surveillance system for tracking the distribution of new HIV-1 genetic variants will be necessary.

## Materials and Methods

Ethics approval has been obtained from the Institutional Review Board of the University of Hong Kong/Hospital Authority (Hong Kong West Cluster) with Reference number UW08-070.

### Patient samples

This study included the first available plasma samples isolated from 1045 HIV-1 positive patients who had used the genotyping resistance testing service of the Department of Health between 2003 and 2008. All patient samples were reported to be treatment naïve at the moment of collection.

### HIV sequencing and phylogenetic analysis

The HIV-1 *pol* sequences were amplified and sequenced by ViroSeq HIV-1 Genotyping System version 2.0 (Celera Diagnostics, CA) or an in-house genotyping method as described previously [8]. All the *pol* sequences incorporated entire protease (297 base pairs; 99 codons) and partial reverse transcriptase (828 base pairs; 276 codons) region, with a total length of 1125 base pairs. The HIV-1 genotypes were then determined by the phylogenetic analysis. The *pol* sequences were aligned with HIV-1 group M 2009 reference sequences in the NCBI Viral Genotyping Tool (http://www.ncbi.nlm.nih.gov/projects/genotyping) by using MUSCLE [16]. Phylogenetic tree was constructed with PAUP* 4.0 using the neighbor joining (NJ) algorithms with 1000 bootstrap replicates [17]. In order to determine the clade assignments and recombinant structure of the genotype unassigned sequences, bootscanning analyses were performed on the genotype unassigned samples with the SimPlot software [18]. The SimPlot performed bootscanning on neighbor-joining trees by using SEQBOOT, DNADIST, NEIGHBOR and CONSENSE from the PHYLIP package on a moving window of 300–400 base pairs along the alignment with 50 base pairs increments. The bootstrap values for the studied sequences were plotted at the midpoint of each window. In the analysis, the new sequences were compared with consensus sequences (50% threshold) representing the HIV-1 variants from the same alignment used for phylogenetic tree analysis.

Furthermore, the *env* C2V3 region of the unassigned samples were amplified and sequenced by 2 pairs of in-house primers (ENV1F 5′-TAGGCATCTCCTATGGCAGGAAGAAGCGG-3′; ENV1R 5′-CACTTCTCCAATTGTCYYTCATATYTCCTCCTCCAGG-3′; ENV2F 5′-ATACATTATTGTGCYCCRGCTGG-3′ and ENV2R 5′-ATGGGAGGGGCATAYATTGC-3′). The 480 base pair sequences were then aligned with *env* sequences of the NCBI HIV-1 2009 reference set.

### Epidemiology data analysis

For determining the epidemiological transmission reservoir of the unassigned genotype samples, the patient epidemiological information including the age, gender, ethnicity, place of birth, route of transmission, plasma sampling date were collected from the Integrated Treatment Centre, Department of Health.

## References

[1] Roberts JD, Bebenek K, Kunkel TA (1988). The accuracy of reverse transcriptase from HIV-1.. Science.

[2] McCutchan FE (2006). Global epidemiology of HIV.. J Med Virol.

[3] Hemelaar J, Gouws E, Ghys PD, Osmanov S (2006). Global and regional distribution of HIV-1 genetic subtypes and recombinants in 2004.. AIDS.

[4] Leitner T, Korber B, Daniels M, Calef C, Foley B (2005). HIV-1 subtype and circulating recombinant form (CRF) reference sequences.

[5] Thomson MM, Najera R (2005). Molecular epidemiology of HIV-1 variants in the global AIDS pandemic: an update.. AIDS Rev.

[6] Zhang Y, Lu L, Ba L, Liu L, Yang L (2006). Dominance of HIV-1 subtype CRF01_AE in sexually acquired cases leads to a new epidemic in Yunnan province of China.. PLoS Med.

[7] Leung TW, Mak D, Wong KH, Wang Y, Song YH (2008). Molecular epidemiology demonstrated three emerging clusters of human immunodeficiency virus type 1 subtype B infection in Hong Kong.. AIDS Res Hum Retroviruses.

[8] Chen JH, Wong KH, Chan K, Lam HY, Lee SS (2007). Evaluation of an in-house genotyping resistance test for HIV-1 drug resistance interpretation and genotyping.. J Clin Virol.

[9] Chen JH, Wong KH, Chan KC, Lam HY, Yuen KY (2008). Molecular epidemiology and divergence of HIV type 1 protease codon 35 inserted strains among treatment-naive patients in Hong Kong.. AIDS Res Hum Retroviruses.

[10] Chen JH, Wong KH, Li P, Chan KC, Lee MP (2009). Molecular epidemiological study of HIV-1 CRF01_AE transmission in Hong Kong.. J Acquir Immune Defic Syndr.

[11] Gueudin M, Plantier JC, Lemee V, Schmitt MP, Chartier L (2007). Evaluation of the Roche Cobas TaqMan and Abbott RealTime extraction-quantification systems for HIV-1 subtypes.. J Acquir Immune Defic Syndr.

[12] Taylor BS, Sobieszczyk ME, McCutchan FE, Hammer SM (2008). The challenge of HIV-1 subtype diversity.. N Engl J Med.

[13] Chen YJ, Huang YH, Chuang SY, Kao DY, Lan YC (2010). Molecular epidemiology of HIV-1 subtype B, CRF01_AE, and CRF07_BC infection among injection drug users in Taiwan.. J Acquir Immune Defic Syndr.

[14] Xiridou M, van Griensven F, Tappero JW, Martin M, Gurwith M (2007). The spread of HIV-1 subtypes B and CRF01_AE among injecting drug users in Bangkok, Thailand.. J Acquir Immune Defic Syndr.

[15] Virtual AIDS Office of Hong Kong (2009). http://www/info.gov.hk/aids.

[16] Edgar RC (2004). MUSCLE: multiple sequence alignment with high accuracy and high throughput.. Nucleic Acids Res.

[17] Saitou N, Nei M (1987). The neighbor-joining method: a new method for reconstructing phylogenetic trees.. Mol Biol Evol.

[18] Lole KS, Bollinger RC, Paranjape RS, Gadkari D, Kulkarni SS (1999). Full-length human immunodeficiency virus type 1 genomes from subtype C-infected seroconverters in India, with evidence of intersubtype recombination.. J Virol.

